# Food Acquisition through Private and Public Social Networks and Its Relationship with Household Food Security among Various Socioeconomic Statuses in South Korea

**DOI:** 10.3390/nu10020121

**Published:** 2018-01-25

**Authors:** Sohyun Park, Kirang Kim

**Affiliations:** 1 Department of Food Science and Nutrition, Hallym University, Hallymdaehak-gil, Life Science Bldg #8519 Chuncheon-si, Gangwon-do 24252, Korea; sopark@hallym.ac.kr; 2 Department of Food Science and Nutrition, College of Natural Sciences, Dankook University, Dandae-ro, Dongnam-gu, Cheonan-si, Chungnam 31116, Korea

**Keywords:** food security, food acquisition, social networks, social support, socioeconomic status

## Abstract

This study was conducted to understand food acquisition practices from social networks and its relationship with household food security. In-depth interviews and a survey on food security were conducted with twenty-nine mothers and one father in metropolitan areas of South Korea. Many families acquired food from their extended families, mainly participants’ mothers. Between low-income and non-low-income households, there was a pattern of more active sharing of food through private networks among non-low-income households. Most of the low-income households received food support from public social networks, such as government and charity institutions. Despite the assistance, most of them perceived food insecurity. We hypothesized that the lack of private social support may exacerbate the food security status of low-income households, despite formal food assistance from government and social welfare institutions. Interviews revealed that certain food items were perceived as lacking, such as animal-based protein sources and fresh produce, which are relatively expensive in this setting. Future programs should consider what would alleviate food insecurity among low-income households and determine the right instruments and mode of resolving the unmet needs. Future research could evaluate the quantitative relationship between private resources and food insecurity in households with various income statuses.

## 1. Introduction

Food insecurity refers to the inability to afford nutritionally adequate and safe food for maintaining a healthy life [1] and it is a growing concern worldwide [2]. Food insecurity has been found to be associated with various conditions, including chronic disease [3], obesity [4,5,6], undernutrition [7,8] and risk of depression and anxiety [9]. Factors that are associated with food insecurity include low household income, low education level, living alone, unemployment, distance traveled to purchase food and no home ownership [10,11].

In South Korea, food insecurity is highly prevalent among low-income households (LIHs). Approximately one-fourth of LIHs were classified as food insecure households in one study [10]. Another study in 5 low-income areas in Seoul, showed that more than 60% of the households were food insecure. Among these food insecure households, the intake of energy and other nutrients from animal sources, such as protein, riboflavin, fat and calcium, were lower than that in their food secure counterparts [12].

Various coping strategies have been explored. Participating in the governmental nutrition assistance programs, such as Supplemental Nutrition Assistance Program (SNAP) and Special Supplemental Nutrition Program for Women, Infants and Children (WIC) in the U.S. [13], is one of the most common strategies. In South Korea, most regional governmental agencies support low-income families with instrumental supports, which supply staples (uncooked rice, fermented cabbage-*kimchi*) to eligible households. In addition, regional public health centers provide a “*Nutrition Plus*” program for pregnant and lactating women and young children (equivalent to the WIC program in the U.S.). Children from LIHs receive free school lunches and “*dream tree cards*”, an Electronic Benefit Transfer (EBT) card, which can be used in designated food outlets. Some government-funded after-school programs, although not specifically designed as nutrition assistance programs, also provide hot dinners along with healthy snacks to children from LIHs [14].

Previous studies have found that food insecure families also utilize private social supports to cope with food insecurity [13,15]. The most commonly mentioned private social resources include families, friends and neighbors [15]. Research has suggested that people with food insecurity alleviate the stress and receive a direct solution to the problem by drawing on support from these social resources [16,17]. In spite of the positive effects of social support in coping with food insecurity, others have suggested that constant dependence on others also causes psychological stress [15]. The authors hypothesized that individuals may feel obligation in returning the support when they are not capable of doing so [18].

In a study of the local food environment and social support on rural food insecurity, the authors reported that households with private support were much less likely to be food insecure [19]. However, the study also found that when support was specifically related to food acquisition, receiving food from private networks did not necessarily alleviate household food insecurity. The authors speculated that this may be due to a normalized form of food exchange happening among people within some rural settings, not a targeted behavior among food insecure households [19].

As food exchange among private social networks is a common practice in some rural U.S. communities [19,20], food exchange or food sharing is deeply rooted in the culture of many communities [21]. South Korea also shares this cultural practice, reinforced by its core cultural value, collectivism [22]. Researchers in cultural psychology argue that the initial formations of collective units (such as families, clans and tribes) in humans were through food sharing among hunting and gathering tribes and agrarian communities. People increased efficiency and survival by sharing ability, resources and food sources [23]. In South Korea, where collectivism plays an important role in shaping the traditional culture, sharing and exchanging foods can be a part of food environment. However, food sharing among private social networks in modern Korean society and its association with household food security remain underexplored.

The goal of the larger research project was to examine the components of the home nutrition environment in urban South Korea. For the qualitative phase, which is the focus of the present article, the role of social support and networks in the household food supply were explored. By recruiting households with various socio-economic statuses, the qualitative inquiries examined how these social supports and food sharing impact household food security across various income levels. The specific objectives of the present study were to: (1) explore the types of social support, especially instrumental support (food) among both food secure and insecure families; (2) examine how these social support systems play a role in food security; and (3) suggest ways to improve government food assistance programs in order to increase food security among low-income households, which is presented in the discussion.

## 2. Materials and Methods

### 2.1. Study Design

This qualitative study was part of a formative research project to develop assessment tools for measuring home nutrition environments. In order to understand which aspects of home nutrition environments should be included in the assessment tools, in-depth interviews along with short surveys on basic socio-demographic information of the participants and their family and food insecurity were conducted. In addition, direct observations were carried out on all the food items in refrigerators and food pantries. This manuscript mainly focuses on the results from in-depth interviews, which were employed to explore various food acquisition pathways in households with various socio-economic statuses and perceived food and nutrition environments in the context of urban South Korea.

When developing the interview guides, an ecological perspective based on the bioecological model [24,25] and the model of the home food environment pertaining to childhood obesity suggested by Rosenkranz and Dzewaltowski [26] were used. During the interviews, various topics were discussed, including the accessibility to food markets in the neighborhoods, household nutrition environments, such as food rules at home, food shopping and stocking routines and other food sources that were given by families and other social networks. Among these topics, this places special emphasis on various food acquisition pathways in the participating households and its relationship with food insecurity, which have not been discussed extensively in South Korea. These qualitative findings were connected to the results of the food insecurity questionnaire and participants’ household incomes in order to understand the roles of various food sources in shaping the household food (in)security.

### 2.2. Participants and Recruitment

Using snowball sampling [27], a convenience sample of 29 women and 1 man with children from metropolitan areas in South Korea participated in the interviews. The interviews were conducted between June and September 2015. To recruit participants from various socioeconomic statuses, a flyer with a description of the research and data collection process was distributed to workers at local public health centers and community welfare centers in various regions with different average housing prices. The workers at the public health and community welfare centers were asked to recruit study participants among center visitors based on the inclusion criteria, which was parents with children between 0 and 12 years of age. If potential participants agreed to participate in the interview, the contact information was transferred to the research team. The interview transcripts were analyzed as the interviews continued; therefore, the recruitment was stopped when it was considered that information saturation had been reached, at which point no new information was gathered with new interviews.

### 2.3. Instruments and Measures

All of the interviews and surveys occurred at participants’ houses using an interview guide and questionnaires. The interviews started with a ‘grand tour’ question [27], which asked the general description of their neighborhoods and their houses and moved to more specific questions on their food shopping routines and the household members’ food intake practices. Before the interviews, researchers observed the food items in their refrigerators and food pantries. During the interviews, the detailed sources of each food item were asked. If some food items were not bought for themselves, how they were acquired was also discussed. The key questions in the interview guide are presented in Table 1.

The food insecurity measurement tool from the Korea National Health Examination and Nutrition Survey was used in this study, which was modified from the U.S. Household Food Security Survey Module (HFSSM) [28]. A score of 1 was assigned to affirmative responses indicating food insecure conditions and a score of 0 was assigned to all other responses in each questionnaire. Food insecurity status was defined by the sum of the scores. Households were classified as food secure if the total score was ≤2 and food insecure if the total score was ≥3.

The household income was measured with a multiple-choice question. LIHs were defined as earning less than 120% of the minimum cost of living. In 2015, when the data collection occurred, the monthly minimum cost of living for a household with 4 members was 1.66 million Korean won, which is approximately 1500 U.S. dollars (USD).

### 2.4. Procedures

All one-on-one interviews took 40 min to 1 h to complete after observing and making a list of the food items at home, followed by additional short surveys on basic socio-demographic factors and household food insecurity measurement. Upon the participants’ approval, all the interviews were digitally recorded and transcribed verbatim. The interviewers reviewed the transcripts for accuracy. The participants received monetary compensation equivalent to $45 USD and counseling on food storage tips. The reason for the relatively high amount of compensation was the lengthy process of the entire data collection process for the study, which included at least 90 min of making a complete list of food items in the house. All of the participants signed written consent forms and the study protocol was approved by the Institutional Review Board of Dankook University (IRB No. 2015-04-025-005).

### 2.5. Data Analysis

Thematic analysis was used to analyze the interview transcript [29,30]. The research team reviewed the earlier interview transcripts and identified significant themes regarding food acquisition through various social networks. The team categorized these significant themes to develop a codebook, a guideline and manual for coding the data. Two authors (S.P. and K.K.) coded the data using the codebook and reviewed the codes. Once the coders coded the first three interviews, the research team discussed the coded data and emerging themes. The research team continued the discussion until the final codes and themes were determined for all of the interview transcripts. The analysis process was assisted by the text-based data management program, Atlas.ti (version 1.0 for Mac, Scientific Software Development GmbH, Berlin, Germany, 2016).

## 3. Results

### 3.1. General Characteristics of the Study Participants

The overall description of the study participants was presented in Table 2. Among 30 participating households, 12 were classified as low-income households. The majority of participating households (27 out of 30) stated that they receive food items from various sources regularly or irregularly. For both low-income and non-low-income households, receiving or sharing foods is common. The sources include family members, friends, governmental agencies, social welfare centers and religious institutions. We categorized receiving foods from family members and friends as ‘private food sharing (assistance)’ and governmental, social welfare, food banks, religious institutions’ support as ‘public food assistance’. For convenience, we use private food sharing and assistance interchangeably in the manuscript because the purpose of sharing food is either to help or simply for sharing within private social networks.

### 3.2. Private Food Sharing and Public Food Assistance

Among 27 households receiving food support, the majority (23 households) reported food sharing within private networks regardless of their income status (6 out of 12 low-income households and 17 out of 18 non-low-income households). Among 12 low-income households, 5 receive both private and public food assistance and 2 receive neither. More details on the frequency of private and public food assistances are provided in Table 2.

### 3.3. Sources and Types of Instrumental Support from Private Social Networks

The first type of instrumental support from private networks was receiving food items from those who grow or prepare them, mostly participants’ mothers or mothers-in-law. In some cases in which the participants were young mothers, they reported receiving foods from their siblings or grandmothers. The most common food items shared by these family members were *kimchi* and basic ingredients or spices for Korean dishes, such as soy sauce, soybean paste, red pepper paste and red pepper powder. Some households mentioned that they also receive grains, uncooked vegetables (green leafy and root vegetables, vegetables for spices, such as pepper, onions, garlic and green onions) and pickled vegetables.

My mother-in-law grows vegetables in her little farm. During the summer, we get pepper, zucchini and bell pepper from her farm. If there’s too much, we share those with my mom.

—Food secure, non-low-income household, a stay-at-home mother with a toddler

My mother has a little farm that grows rice, vegetables, potatoes, sweet potatoes and pepper… So basically, all the basic spices and seasonings came from my mom. She sent us periodically, not during the winter though. And she also sends us sesame seed oil, which she makes.

—Food secure, non-low-income household, working part-time with three children

The second type of private sharing was receiving ready-to-eat foods from extended family members. Families with young children mentioned this type of support more frequently. This type of food support can be regarded as another form of childcare support because mothers with young children can save time on food preparation and pay more attention to childcare. Participants with school-aged children said that they did get foods from their mothers more often when the children were younger, especially prepared dishes that are ready to eat rather than raw ingredients or *kimchi* only.

Since I have a little baby, my parents-in-law think that we don’t have much time and energy to cook and eat. So, when we meet at the church every Sunday, my father-in-law brings all sorts of things… even they bring a pot of soup… the weather’s hot nowadays, so they worry that I cannot cook that much. They take care of us so much. And my mother lives nearby and now she stays home after retiring from work, she brings anything whenever I need.

—Food secure, low-income household, stay-at-home mother with one child

My family visits my mother’s and mother-in-law’s place every other week. So, mothers prepare a lot of food for the children. We usually have big dinners and bring leftovers… bring many different things…

—Food secure, non-low-income household, stay-at-home mother with two children

In addition to prepared foods, some participants mentioned that they receive certain food items that are relatively easy to prepare, such as marinated meat dishes or fish.

My mother-in-law bought rice and snacks for my kids. She also brings vegetables, bulgogi (marinated beef), mackerel, cutlassfish and different kinds of fish…usually at least once every two weeks.

—Food secure, non-low-income household, stay-at-home mother with two children

Some households received more than just basic ingredients. Cookware, chinaware, or packaged foods that children like (such as milk, snacks and canned foods) were also mentioned, although the number of these households was not large (3 out of 27 households).

The third type of private food sharing is among friends or neighbors. Mothers often have a circle of friends through their children and they mentioned “*buy foods from warehouse club, such as Costco or online farmers’ market in large quantities and share them with friends*”. By doing so, they can get fresh produce or quality meat in bulk at cheaper prices.

### 3.4. Sources and Types of Instrumental Support from Public Social Networks

All the participants who mentioned that they receive instrumental support from public social networks were categorized as LIHs. Most of them qualified for government assistance programs, such as the *Nutrition Plus* program, in which qualified mothers and children receive nutrition education and food items biweekly from regional public health centers. Some of these households also reported that they receive basic food items, mostly rice and *kimchi*, from the social welfare divisions of provincial or local public offices. In addition to the government agencies, food banks, local churches and social welfare centers operated by private institutions were mentioned as their social networks for instrumental food support.

As opposed to various food items mentioned through private social networks, limited varieties were listed for the types of public support. As mentioned earlier, uncooked rice and *kimchi*, which are the Korean staples, are the most common items provided by public agencies. Occasionally, food lists include prepackaged foods, such as dry noodles, instant ramen noodles, flour and factory-made sauces.

Participants also said that some social welfare centers have special after-school programs offering fruits or hot meals to their school-aged children. Two of the participants mentioned that local social welfare centers run by religious organizations offer food delivery with hot meals a few times per week. Lastly, low-income families with school-aged children receive debit cards called “*dream tree cards*” from the government, with which the families can buy milk or other food items for their children at designated food markets.

### 3.5. Perceptions and Satisfaction of Food Support from Public Social Networks among Low-Income Households

During the interviews, we probed further into the participants’ perception of the food assistance from public social networks. Generally, participants with public assistance were not satisfied with the help. The food lists lack fresh produce, and, with the exception of the hot meal delivery, most of the foods given were basic ingredients, which require cooking. Due to busy work and childcare schedules, mothers from low-income families do not find these items as useful in their families’ diet. Moreover, families who qualify for government assistance tend to have two or more children and still find it hard to feed them sufficiently with their income and the instrumental support from public social networks.

We buy milk with the food card. Since there are four kids, we cannot really afford that much. They never get to eat enough. If we buy six cartons of milk, about 6 liters of milk, they are gone in two days. I feel very sorry and sad that I can’t provide for them.

—Food insecure, low-income household, stay-at-home mother with four children

We get food from the food bank. But the problem with the foods from the bank is that there is so much that we can’t really use. There is a limited selection of food items. And they give the same varieties in large quantities. We get the same things over and over again… what I really need is basic seasonings, such as soy bean sauce, red pepper paste and vinegar and so on. I can understand that vegetables can go bad quickly but why don’t they provide potatoes or sweet potatoes? That would be so nice.

—Food insecure, low-income household, stay-at-home mother with four children

On the other hand, there was also some positive feedback. Parents whose children attend after-school programs at local welfare centers were satisfied with the healthy snacks and hot dinners. Since they do not have to cook dinners for them after work, it saves their time and resources. Parents also think that these hot dinners are “*good for the kids’ diet, since they get to eat various vegetables and fruits*”. One single mother said that she used to receive hot meal boxes or fruit boxes from a local social welfare center when her daughter was in middle school. Now that her daughter is in high school, she is not qualified for the free meal delivery or fruit boxes any longer. Since she has higher expenditures due to her daughter being in high school, it has become more difficult for them to buy healthy food items, such as fruits.

When my daughter was in middle school, she brought a pack of fruits from the after-school program. And I used to buy fruits that were on sale… but not anymore. Because she (daughter) is now in high school, the expenses for her have gone up and we can’t really afford to buy fruits. We used to get hot meals when she was younger but not anymore. Heard that we are not qualified any longer.

—Food insecure, low-income household, a working single mother with one child

### 3.6. Private and Public Food Assistance and Household Food (in)Security

In-depth interviews were analyzed with the results from the household food security survey. The results showed that sharing or receiving food among private social networks was more common among non-LIHs. Most of the non-LIHs (94.4%) participated in private food sharing, whereas only 50.0% of LIHs participated in food sharing (Table 2). Among 17 food secure households, 16 households reported that they have private food assistance and all of them were non-LIHs. Among 13 food insecure households, only 7 households had occasional private food assistance. In addition, among these 13 food insecure households, 10 households had public food assistance but still regarded themselves as food insecure.

The interviews show that food insecure households did not have a sufficient monthly income, which makes it difficult for the families to spend enough money on food. Most of these low-income and food insecure households reported that “*food cost is always burdensome*”. In addition to the lack of monetary resources, some of these households lack private social resources and food assistance through these resources, compared to food secure households with a relatively higher income. It can be hypothesized that this lack of social resources through private networks may exacerbate their household food insecurity.

Interviewer: Do you have anyone who can help or share some foods? How about mother?

Interviewee: No… I haven’t had mother for a long time. I am used to it. It’s tough but it is the way it’s been. I am used to it.

—Food insecure, low-income household, a working single mother with one child

Among 10 LIHs with either public or private food assistance, 9 households still evaluate themselves as food insecure households. Moreover, among 5 LIHs with both public and private food assistance, 4 households regarded themselves as food insecure. Based on the interviews, with the limited variety of foods provided by public assistance, which are restricted to uncooked rice, *kimchi* or some packaged foods that are not attractive, the interviewees in LIHs always feel scarcity, especially regarding fresh vegetables, fruits and animal-based protein sources (fish and meat). These food items are more expensive than basic staples in South Korea. Therefore, with private and public assistance, many LIHs still perceive that they do not have enough food to feed their family.

The quote below illustrates how important private food sharing can be in assuring food security among families with children by saving food costs.

I don’t think we spend that much money on foods… compared to other households. Because when I talk to other mothers in the neighborhood, their expenses are a lot more than mine. Since I have a mother and mother-in-law, who send various things occasionally… such as soy sauce, red pepper paste…I don’t need to spend money on those things.

—Food secure, non-low-income household, stay-at-home mother with two children

In addition, low-income families often reported that they cannot afford eating out or delivery foods, such as fried chicken, which is a popular delivery food item in South Korea, especially among children. Interviewees felt deprived because they cannot provide these delivery foods when their children ask for them. While it is not clear whether this psychological deprivation can also be connected to nutritional deprivation with the current data, it may exacerbate perceived food insecurity among LIHs.

My daughter eats dinner at 5 at the afterschool program. So, when she comes back home around 8 or 9, she asks for something…like fried chicken. Then I say we’ve got no money… then she feels frustrated that… we can’t even afford fried chicken. I am usually heartbroken that I can’t provide enough for her. Last time, we couldn’t even pay the rent for two months. So it’s tough.

—Food insecure, low-income household, a working single mother with one child

On the other hand, non-LIHs with food security mentioned occasional eating out and delivery food intake in addition to various sources of food sharing, such as extended families, neighbors and friends.

Moreover, older mothers with older children and larger families in LIHs were in worse situations. Most of the participants in their 40s and 50s did not have any parental support and had older children in middle or high schools. They tended to have more regular expenditures on housing and children’s education; therefore, they perceived more food deprivation.

## 4. Discussion

We examined food acquisition practices through private and public social networks and its relationship with food security among urban households with children across various income statuses in South Korea. There was a clear pattern that non-LIHs with children had more active food sharing with their families (mainly with mothers), compared to their low-income counterparts. With higher income status and ample food support from private networks, non-LIHs were more food secure. On the other hand, participants from LIHs, with limited social networks other than support from governmental and community supports suffered more from food insecurity. 

Previous studies from the U.S. and Canada have shown that one of the main strategies for coping with food insecurity is drawing on social support from private and public assistance [13,15,16,31]. Family, friends and neighbors are the main sources of private (informal) social support among low-income families in rural America [15]. Community institutions, such as faith organizations, food banks and governmental safety net programs, such as the Supplemental Nutrition Assistance Program (SNAP), previously called the Food Stamp Program (FSP) and the Special Supplemental Nutrition Program for Women, Infants and Children (WIC), were the most frequently discussed public (formal) sources for alleviating food insecurity among U.S. LIHs [13].

Participants of this study mentioned similar sources for their food acquisition. Unlike previous studies, however, this study includes non-low-income and food secure households and found that these non-LIHs had active food sharing, mostly receiving foods from their mothers and mothers-in-law. The larger project that the current data derived from was to understand various elements in home food environment in urban South Korea. We considered that these food items received from extended family in the urban setting could be an important element that shapes household food availability and affects how the family feels about their food security. The role of private resources on food insecurity shown in this study, however, might be different from rural families where food production within the household is common. As shown in the Results, many interviewees in the urban areas had extended families that grow food in rural areas. Moreover, rural areas traditionally had tighter social networks with neighbors [32]. With prevalent food production and closer relationship with social networks based on geography, food acquisition practices through social networks may have different characteristics and impact on household food insecurity. This issue needs to be further explored with future studies focusing on association between food sharing and food insecurity in rural areas.

Social support and social capital were emphasized as key factors in supporting communities’ and individuals’ health and general well-being [33,34]. There has been some discussion that links social support to nutritional status. According to Locher et al. (2005), perceived lack of social support is associated with increased nutritional risk among the older population [35]. Previous studies on social support and its role in food insecurity have were mainly been undertaken in the rural areas of the U.S. [15,16]. To our knowledge, this is the first study to examine food acquisition practices through social networks and its relationship with food insecurity in households with children in Asian urban settings. 

Food sharing is deeply rooted in Asian culture [36] and it has been found that sharing various food items with adult children is common in South Korea. Using the interview data, a clear pattern was found that non-LIHs had richer informal social networks that share various food items. On the other hand, the proportion of LIHs that have active food sharing practices within private social networks was less than their non-LIH counterparts. It was hypothesized that this lack of private social networks, coupled with the lack of income that can be spent on foods, may exacerbate food insecurity for LIHs. This hypothesis is supported by a U.S. study, which shows that informal support was more helpful in coping with food insecurity among rural households, compared to formal governmental support [19]. Future studies with a larger sample size should examine this relationship further. This relationship may even provide a deeper understanding of the polarization of wealth and intergenerational transfer of poverty.

Previous studies from the U.S. and Canada have presented dissatisfaction expressed by the recipients of public nutrition assistance. Most of the recipients still experienced food insecurity regardless of the assistance. The authors discussed that the government food assistance is not sufficient to alleviate food insecurity among low-income participants, or the participants do not have enough skills in purchasing and cooking the available food provided by the government [13,37,38]. A previous study conducted in South Korea also showed that food assistance programs did not alleviate household food insecurity [10].

The low-income participants from this study shared similar experiences. In particular, households with multiple children often encountered children’s wants and needs, which are not met by their own purchasing power and governmental support. Even with familial support, some of the families perceived food insecurity because they cannot afford food shopping with their own income and they are not satisfied with the food items provided by their families. The most commonly mentioned food items that they felt were lacking included animal-based protein sources, fruits and vegetables during the winter seasons, all of which are relatively more expensive than other food items in this study setting. Moreover, despite the needs, these food items are not commonly supplied by public food assistance. In South Korea, the most common food items provided by the government and social welfare institutions are uncooked rice and *kimchi.* A contribution of this study is greater understanding of why people with public nutrition assistance still perceive food insecurity.

It is noteworthy that some targeted nutrition programs, such as providing hot dinners and fruit baskets for school-aged children, run by some local after-school programs, received positive feedback from the recipients. Future public food assistance programs should be tailored more precisely to meet the potential participants’ unmet needs and wants.

The participants of this study were parents. We found that, especially for the families with infants and toddlers, the extended families could be the major source of the family’s food supply. In that case, these informal food supports can play an important part in shaping the household food environment by alleviating the financial and time burdens to prepare meals while taking care of young children. Not everyone among the study participants with young children had this type of support. Some mothers from LIHs did not have instrumental support from extended families and they were constrained both by income and time due to childcare, which affected household food insecurity.

Despite of the contribution of this study, there are some limitations. Data on home ownership or the proportion of rental fees within the households’ monthly expenditure were not collected. For many households, housing expenses are one of the biggest expenditures and impacts their disposable income for food and food insecurity [10]. De Marco et al. mentioned that home ownership is one of the primary avenues through which low-income families can build equity and gain tax advantages [13].

Representativeness of study participants is not a main concern of qualitative studies. However, when interpreting the study results, one should consider the larger part of this study, which involved documenting all the food items in the household. Therefore, the burden of study participation was more than just filling out survey questionnaires and interviewing. Parents with more free time and willingness to open up their pantry to research teams may be different from all urban families in this study setting.

Given the prevalent practices of food sharing in this setting, nutrition assistance programs can be developed based on this cultural aspect. Instead of trying to provide everything, governmental agencies may design community-based participatory programs that can provide a platform to connect low-income families to other families with more resources, time and willingness to share extra foods with neighbors in need. As most mothers in South Korea are willing to share their foods with their adult children, families with young children can benefit from their older neighbors with more resources.

This idea is similar to the recent housing program, called ‘home sharing’ in the City of Seoul, which connects older people with extra rooms to college students who need a room in the city [39]. Older people can earn extra income from the rent and alleviate social isolation, while college students can get a place at less than half of the regular rate in the city. This win-win strategy can be applied to programs for improving urban food security. Tax benefits or other incentives for using public services can be given to the sharers.

From the interviews, most of the participants receiving public nutrition assistance were dissatisfied with the limited selection of food, including the lack of fresh produce and animal-based protein sources. On the other hand, farmers and retail markets dispose of enormous amount of edible foods [40,41,42] because the shape or color of produce is imperfect or the shelf lives are too short. Therefore, making use of unsold food could be one solution to improve food security among urban LIHs and to reduce food waste. Recently, France enacted a law that bans chain supermarkets from throwing away foods [43]. More time is needed to see the impact of this law on the food security of the community [44]; however, reducing food waste is a high priority among other countries [45,46]. By donating unsold food to low-income members of the community, food providers may get tax incentives or reimbursement for the transportation fees for encouraging participation.

Lastly, for low-income families with young children, government programs can consider providing hot meals for the family or weaning foods for babies that can be readily fed. Time, income and the burden of childcare are all intertwined and providing what the families need most will increase the impact of the policy and programs.

## 5. Conclusions

Using a short survey on food security and in-depth interviews, this study showed that many families with children, regardless of their income status, acquired food from their extended families, friends and neighbors. The main source of informal food sharing was participants’ mothers. Between LIHs and non-LIHs, there was a pattern of non-LIHs having more active sharing of food among private social networks. Most of the LIHs received food support from public social networks, such as government and charity institutions.

Despite the assistance, most LIHs perceived food insecurity. Interviews revealed that certain food items were still lacking, such as animal-based protein sources and fresh produce. However, hot meals and fruit baskets for school-aged children received positive feedback. Future programs and policies should consider what would most effectively alleviate the food insecurity among LIHs with children and find the right instruments and mode of resolving the unmet needs and desires.

## Figures and Tables

**Table 1:** Key questions in the in-depth interview guide for understanding food acquisition from private and public social networks in South Korea.

Question Number	Questions
1	Please describe your neighborhood. What would I see if I walked around this neighborhood? Probing on the food outlets (supermarkets, traditional open markets)
2	Please describe your home. How would you describe your kitchen and utensils?
3	Please tell me about food purchasing or acquisition practices. Probing on the food sources, when, who, where to buy or get foods. Ask them to articulate private or public social support related to food acquisition.
4	Describe the most common day of the week and weekend. Please tell me about your household members and their general eating practices. Probing on food preparation and food delivery. Probing on other types of food preparation during weekdays and weekends. Probing on difficulties or concerns in preparing meals for the family.

**Table 2:** The overall description of study participants for understanding food acquisition from private and public social networks in South Korea.

	Non-Low-Income Households (*n* = 18)	Low-Income Households * (*n* = 12)	Total (*n* = 30)
Women (*n*)	18	11	29
Age in years (mean, range)	38.8 (29–50)	39.8 (26–51)	39.2 (26–51)
Region of residence (*n*)			
Seoul	14	10	24
Gyeonggi-do	3	2	5
Others	1	0	1
Education (*n*)			
Less than high school	0	2	2
High school graduates	3	7	10
College graduates and above	15	3	18
Monthly income (*n*)			
<1,000,000 KRW	0	2	2
1,000,000–2,000,000 KRW	0	9	9
2,000,000–3,000,000 KRW	7	1	8
3,000,000–4,000,000 KRW	4	0	4
>4,000,000 KRW	7	0	7
Number of household members (mean, range)	4.1 (3–5)	4.2 (2–7)	4.1 (2–7)
Having private food sharing (*n*, %)	17 (94.4%)	6 (50.0%)	23 (76.7%)
Having public food assistance (*n*, %)	5 (27.8%)	9 (75.0%)	14 (46.7%)
Having both private and public food supports (*n*, %)	5 (27.8%)	5 (41.7%)	10 (33.3%)
Having no food supports	1 (5.6%)	2 (16.7%)	3 (10.0%)
Food insecure household (*n*, %)	2 (11.1%)	11 (91.7%)	13 (43.3%)

* Criteria for low-income households: monthly household income less than 120% of the minimum cost of living in 2015; for example, the minimum cost of living for 4 household members in 2015 was 1.66 million Korean won, or approximately 1500 USD.
